# PET/MRI Radiomics in Patients With Brain Metastases

**DOI:** 10.3389/fneur.2020.00001

**Published:** 2020-02-07

**Authors:** Philipp Lohmann, Martin Kocher, Maximillian I. Ruge, Veerle Visser-Vandewalle, N. Jon Shah, Gereon R. Fink, Karl-Josef Langen, Norbert Galldiks

**Affiliations:** ^1^Institute of Neuroscience and Medicine (INM-3/-4/-11), Research Center Juelich, Jülich, Germany; ^2^Department of Stereotaxy and Functional Neurosurgery, Faculty of Medicine and University Hospital Cologne, University of Cologne, Cologne, Germany; ^3^Center of Integrated Oncology, Universities of Aachen, Bonn, Cologne, and Duesseldorf, Cologne, Germany; ^4^JARA-BRAIN-Translational Medicine, Aachen, Germany; ^5^Department of Neurology, Rheinisch-Westfälische Technische Hochschule (RWTH) Aachen University, Aachen, Germany; ^6^Department of Neurology, Faculty of Medicine and University Hospital Cologne, University of Cologne, Cologne, Germany; ^7^Department of Nuclear Medicine, Rheinisch-Westfälische Technische Hochschule (RWTH) Aachen University, Aachen, Germany

**Keywords:** artificial intelligence, machine learning, deep learning, brain tumors, textural features, amino acid PET, CT

## Abstract

Although a variety of imaging modalities are used or currently being investigated for patients with brain tumors including brain metastases, clinical image interpretation to date uses only a fraction of the underlying complex, high-dimensional digital information from routinely acquired imaging data. The growing availability of high-performance computing allows the extraction of quantitative imaging features from medical images that are usually beyond human perception. Using machine learning techniques and advanced statistical methods, subsets of such imaging features are used to generate mathematical models that represent characteristic signatures related to the underlying tumor biology and might be helpful for the assessment of prognosis or treatment response, or the identification of molecular markers. The identification of appropriate, characteristic image features as well as the generation of predictive or prognostic mathematical models is summarized under the term radiomics. This review summarizes the current status of radiomics in patients with brain metastases.

## Introduction

Brain metastases are one of the most common neurological complications of extracranial cancer and account for more than half of all brain tumors (1). In patients with solid cancers, the risk to develop brain metastases depends on the type and initial stage of the primary tumor. It is in the range of 5–20% and may be increasing due to improvements in control of extracerebral disease by modern systemic treatment and the resulting increasing life expectancy, and technical advances in medical imaging for the detection of small brain metastases (1–3).

Lung cancer, breast cancer, and melanoma are the most common primary tumors that lead to the formation of brain metastases in adults and account for 67–80% of all cancers (1). In about 10% of patients with brain metastases, the primary tumor is unknown (cancer of unknown primary, CUP) (1, 4). Standard treatment for patients with oligometastatic brain disease includes surgical resection, radiotherapy (predominantly stereotactic radiosurgery), and combinations thereof (5). Whole-brain radiotherapy is frequently used in patients with multiple brain metastases. Furthermore, modern systemic treatment options such as immunotherapy including checkpoint inhibitors and targeted therapy are increasingly used to control intra- and extracranial disease (6, 7). Importantly, some molecularly defined subgroups of patients have been identified which have an improved prognosis (8) and benefit from these recently developed agents, e.g., combined BRAF/MEK inhibition using the kinase inhibitors dabrafenib plus trametinib in patients with BRAF-mutant melanoma brain metastases (9).

Magnetic resonance imaging (MRI) is the method of choice to evaluate patients with brain lesions such as primary or metastatic brain tumors. MRI offers excellent soft tissue contrast and a high availability, but its specificity is low (10–12). For example, contrast-enhancing lesions during follow-up and signal alterations on T2 or fluid attenuated inversion recovery (FLAIR) MRI may be non-specific and can result from various causes other than tumor tissue such as infection, demyelination, inflammation, ischemia, or treatment-related changes after surgery, radiotherapy, or systemic therapy. Consequently, with the use of conventional MRI alone, important diagnostic challenges remain such as the differentiation of local brain metastasis relapse from radiation injury and the evaluation of response to treatment that included immunotherapy (13, 14). The latter may lead to the clinically important phenomenon of pseudoprogression, which is characterized by worsening imaging findings on conventional MRI during follow-up caused by treatment-related changes imitating tumor progression that spontaneously vanish during further follow-up without treatment (14). A false diagnosis of pseudoprogression carries the risk of a premature termination of an effective treatment with serious consequences for the patients (13–15).

Advanced MRI techniques have been introduced in the last years to overcome some of the aforementioned limitations of conventional MRI in patients with brain tumors. The advanced MRI techniques currently under investigation in neuro-oncology include, but are not limited to, diffusion- weighted imaging (DWI), perfusion-weighted imaging (PWI), and MR spectroscopy (MRS) (16–19). These techniques might complement conventional MRI by providing insights into additional tumor characteristics such as perfusion, angiogenesis, cellularity, pH, or metabolite concentrations beyond anatomical information.

Another advanced imaging method extensively evaluated in neuro-oncology is positron emission tomography (PET) with tracers other than the traditionally used 2-[^18^F]-fluoro-2-deoxy-D-glucose (FDG) for the characterization of tumor metabolism. It has been emphasized by the Response Assessment in Neuro-Oncology (RANO) working group, the European Association for Neuro-Oncology (EANO), and the Society for Neuro-Oncology (SNO) that the additional clinical value of amino acid PET tracers such as [^11^C]-methyl-L-methionine (MET), O-(2-[^18^F]fluoroethyl)-L-tyrosine (FET), or 3,4-dihydroxy-6-[18F]-fluoro-L-phenylalanine (FDOPA) in patients with gliomas (11, 20, 21) and also brain metastases (4, 22) is outstanding and superior to FDG for various clinical indications.

Although a variety of imaging modalities are used or currently being investigated for patients with brain tumors including brain metastases, clinical image interpretation to date uses only a fraction of the underlying information. Importantly, the images contain complex, high-dimensional digital information that can be made accessible by means of advanced image analysis using machine learning techniques. The growing availability of high-performance computing allows the extraction of quantitative imaging features from medical images that are beyond human perception. Using machine learning techniques and advanced statistical methods, subsets of these imaging features are used to generate mathematical models that represent characteristic signatures related to the underlying tumor biology and might be helpful for the assessment of prognosis or treatment response, or the identification of molecular markers. The identification of appropriate, characteristic image features as well as the generation of predictive or prognostic mathematical models is summarized under the term radiomics (23–26).

Radiomics is usually applied in standard-of-care medical images from any imaging modality (e.g., CT, MRI, PET), thereby allowing additional data evaluation at low cost. The computed radiomics features, either predefined (feature-based radiomics) or generated by supervised learning (deep learning-based radiomics), are more reliable, robust, and reproducible compared to the visual interpretation of imaging features, because radiomics features are computed semi- or fully-automatically.

Another application of radiomics analysis, radiogenomics, aims at the prediction of molecular biomarkers such as genetic mutations, chromosome alterations, or methylation profiles from image data (27). Typically, such biomarkers require tissue samples obtained by stereotactic biopsy or tumor resection and are not accessible by conventional, qualitative image analysis. Consequently, radiogenomics as a non-invasive method to assess biomarkers in patients with brain tumors is of great scientific and clinical interest.

This review summarizes the current status of radiomics in patients with brain metastases.

## Radiomics

Basically, radiomics can be subdivided into feature-based and deep learning-based radiomics. Feature-based radiomics uses mathematically predefined image features that are extracted and computed from preprocessed and segmented medical images. Using machine learning techniques, a subset of these features is selected for the generation of a predictive or prognostic model related to the research question.

Deep learning-based radiomics is fundamentally different as it does not require image segmentation or pre-defined features. In deep learning, artificial neural networks imitate the function of the human visual system and automatically extract high-dimensional features from the original images at different abstraction levels and such autonomously learn characteristic patterns and classify them. In the following section, the basic principles of feature- and deep learning-based radiomics image analysis are briefly introduced.

### Feature-Based Radiomics

#### Pre-processing

Radiomics aims at the extraction of quantitative features from medical images (23–26). Consequently, the imaging data supposed to be analyzed have to be quantitative or at least semi-quantitative. In order to allow reproducible and comparable results, especially if data from different scanners or acquisition protocols are used, an upfront normalization procedure is necessary which may include intensity normalizations, spatial smoothing or re-sampling, other types or image filtering or corrections of MRI field inhomogeneities (24, 28–30).

#### Segmentation

For brain metastases, segmentation is usually performed manually on conventional MRI or CT. Although brain metastases are usually well-circumscribed contrast-enhancing lesions, the manual, three-dimensional segmentation is time-consuming. To overcome this issue, machine learning techniques are being developed for the automated detection and segmentation of brain metastases using deep learning (31, 32). However, these tools still have to prove their reliability and added value to ultimately become part of clinical routine.

#### Feature Extraction

Different kind of quantitative features can be extracted from medical images, which are usually grouped into the following subgroups:

- *Shape features*: Geometric properties of the segmented region of interest (ROI) or volume of interest (VOI) such as compacity, sphericity, volume, or maximum surface can be described by shape features.
- *Histogram-based features* (first-order statistics features): Histograms are used to characterize the distribution of individual voxel intensity values within the ROI or VOI without considering their spatial orientation. From the histogram, measures such as the mean, median, minimum, maximum, entropy (randomness), uniformity, asymmetry (skewness), or kurtosis (flatness) can be calculated.
- *Textural features* (second-order statistics features): The intra-tumoral heterogeneity can be quantified by means of textural feature analysis. Textural features represent statistical relationships between intensity of neighboring voxels and groups of voxels. Textural features are not directly calculated from the image, but from special matrices that already represent a certain aspect of intravoxel relationship; i.e., the gray-level co-occurrence matrix (GLCM) represents the incidence of voxels with the same intensity values at a certain distance along a fixed direction. Another frequently used matrix, the gray-level size-zone matrix (GLSZM), represents the distribution of groups of voxels with the same intensity. Several other matrices exist from which a number of different textural features can be calculated (33).
- *Higher-order statistics features*: Features extracted by statistical methods after the application of mathematical transformations (filters) for, e.g., edge enhancement, noise suppression, or the identification of repeating patterns or histogram-oriented gradients are considered higher-order statistics features. Such mathematical transformations or filters include Laplacian transforms of Gaussian-filtered images (Laplacian-of-Gaussian, LoG), wavelet transforms, fractal analysis, or Minkowski functionals.

In this way, hundreds to thousands of quantitative features can be extracted from a single medical image.

#### Feature Selection and Model Generation

As mentioned above, hundreds to thousands of features can be easily extracted from a single medical image, which is why the relevant parameters from the large number of available features have to be extracted. This essential step is called feature selection (26).

Once a subset of important features is identified, a mathematical model can be generated that predicts the known, underlying ground truth such as a certain genotype or a better prognosis. Commonly used machine learning algorithms for model generation in radiomics are decision trees (e.g., random forests), linear or logistic regression, support vector machines, and k-nearest neighbors. These algorithms are tested for classification accuracy in a subset of data (training dataset). Then, in order to assess the robustness of the model, the best-performing model is applied to another subset of data that were not used during the process of model generation (validation dataset). Ideally, the model is finally applied to a third dataset (test dataset) including imaging data acquired from different institutions using different scanners and different acquisition protocols in order to evaluate the generalizability of the model. However, these steps require large amounts of data (e.g., 70% of images for training/validation and 30% for testing).

In cases in which the number of patients is small and no reasonable and balanced data splitting into a training and a test cohort can be performed prior to model generation, statistical methods such as cross-validation can be applied to estimate the model performance without the availability of a test dataset. The available datasets are partitioned into *k* subsets of equal size, and one subset is retained as testing data, while the remaining *k*-1 datasets are used as training data. Afterwards, the process is repeated *k*-times with each subset used once as testing data. The model performance estimators from each *k* iteration can then be averaged to produce a single estimation of model performance.

### Deep Learning-Based Radiomics

Deep learning as another sub-category of machine learning or artificial intelligence uses artificial neural networks that simulate the neural structure of the brain for classification of high-dimensional non-linear data or pattern recognition (34).

Conventional machine learning algorithms require a workflow involving image preprocessing, segmentation of the ROI, and definition of the inherent features using feature selection techniques followed by model generation and validation. Artificial neural networks automatically extract high-dimensional features from the original or preprocessed images at different scaling and abstraction levels, and autonomously learn the patterns and classify them (35). A cascaded system of single layer neural networks is trained to identify and learn relevant structures within the image data that are useful for classification without any prior definition or selection. These complex structures are then combined to generate features with a higher level of abstraction. The output from the very last layer of the network is then used to fit a prediction model.

However, artificial neural networks strongly depend on the input data and usually require large amounts of image for the identification of robust and representative features which limits its applicability in neuro-oncological research, where the number of available datasets usually is small. One technique to overcome this issue is called transfer learning, wherein an artificial neural network is utilized that was already trained for a different, but similar task; e.g., a neural network that has been used for the classification of glioma subtypes might also be useful for the classification of brain metastases (36). Thereby, the amount of data necessary for training the network can be reduced since the network already has some prior knowledge about brain lesions.

## Radiomics in Patients With Brain Metastases

Radiomics in patients with brain metastases is mainly based on the analysis of conventional MRI data. The majority of studies investigated the usefulness of radiomics to differentiate treatment-related changes from brain metastases recurrence after radiotherapy, which is one of the most important indications in the field. Some studies have also evaluated the value of radiomics for the prediction of brain metastases origin and the differentiation of brain metastases from glioblastoma. Furthermore, radiomics in patients with brain metastases was used for treatment response assessment. In the following, the key findings of radiomics-based research in patients with brain metastases are summarized. An overview of the discussed studies and the main results is provided in Table 1.

**Table 1:** Radiomics based on MRI and/or PET in patients with brain metastases.

**Study**	**No. of patients (patients/lesions)**	**Purpose**	**PET tracer**	**MRI contrast(s)**	**Classification method**	**Validation method**	**Model applied to a separate test dataset?**	**Highest accuracy /Most important result**
Peng et al. (37)	66/82	Differentiation of TRC from BM recurrence	n.a.	T1-CE, FLAIR	Support vector machines	LOOCV	No	0.81 (AUC)
Zhang et al. (38)	87/97	Differentiation of TRC from BM recurrence by delta radiomics	n.a.	T1, T1-CE, T2, FLAIR	Ensemble trees	LOOCV	No	0.73 (AUC)
Lohmann et al. (39)	47/54	Differentiation of TRC from BM recurrence	FET	n.a.	ROC analysis	n.a.	No	85%
Lohmann et al. (40)	52/52	Differentiation of TRC from BM recurrence	FET	T1-CE, T2, FLAIR	Logistic regression	5-fold CV, 10-fold CV, LOOCV	No	89%
Hotta et al. (41)	41/44	Differentiation of TRC from BM recurrence	MET	n.a.	Random forest	10-fold CV	No	0.98 (AUC)
Ortiz-Ramon et al. (42)	30/50	Prediction of BM origin	n.a.	T1	Naive Bayes	Nested CV	No	0.95 (AUC)
Ortiz-Ramon et al. (43)	38/67	Prediction of BM origin	n.a.	T1	Random forest	Nested CV	No	0.96 (AUC)
Kniep et al. (44)	189/658	Prediction of BM origin	n.a.	T1, T1-CE, FLAIR	Random forest	Model-external 5-fold CV	Yes	0.82 (AUC)
Qian et al. (45)	412/412	Differentiation of BM from GBM	n.a.	T1-CE	Support vector machines	5-fold CV	Yes	0.90 (AUC)
Artzi et al. (46)	439/439	Differentiation of BM from GBM	n.a.	T1-CE	Support vector machines	5-fold CV	Yes	0.96 (AUC)
Cha et al. (35)	89/110	Prediction of treatment response to SRS	n.a.	CT only	Ensemble model (CNN)	Validation dataset	Yes	0.86 (AUC)
Della Seta et al. (47)	48/48	Prediction of treatment response to SRS	n.a.	T1-CE	Cox regression	n.a.	Yes	Enhancing tumor volume associated with a 2.1-fold longer OS (*p* = 0.005)
Bhatia et al. (48)	88/196	Prediction of treatment response to immune checkpoint inhibitors	n.a.	T1-CE	Cox regression	n.a.	Yes	Radiomics features associated with prolonged OS (*p* = 0.001)

*AUC, area under the receiver operating characteristic (ROC) curve; BM, brain metastasis; CNN, convolutional neural network; CT, computed tomography; CV, cross-validation; FET: O-(2-[^18^F]fluoroethyl)-L-tyrosine; FLAIR, fluid attenuated inversion recovery; GBM, glioblastoma; HR, hazard ratio; LOOCV, leave-one-out CV; n.a., not available; OS, overall survival; ROC, receiver operating characteristic; SRS, stereotactic radiosurgery; T1-CE, contrast-enhanced T1-weighted MRI; TRC, treatment-related changes*.

### Differentiation of Treatment-Related Changes From Brain Metastases Recurrence

Patients with brain metastases are increasingly treated with stereotactic radiosurgery. Not infrequently, radiation injury (e.g., radiation necrosis) may occur after radiosurgery and is often indistinguishable from actual tumor progression using conventional MRI alone.

Peng et al. (37) evaluated the usefulness of MRI radiomics for this important question. Sixty-six patients with 82 lesions treated with stereotactic radiosurgery and imaging findings on contrast-enhanced T1 and FLAIR sequences suspicious for tumor recurrence were included in the study. Fifty-one radiomics features (3 shape features, 14 histogram-based features, and 34 textural features) were extracted for each lesion on each MRI contrast. Models were generated using the IsoSVM algorithm which performs both feature selection and classification (49). No separate dataset was available for model testing. However, cross-validation was performed to assess overall model performance. The model reached an area under the receiver operating characteristic curve (AUC) of 0.81 with a specificity of 65% and a sensitivity of 87%. On the contrary, experienced radiologists could only classify 73% of the cases with a sensitivity of 97% and a specificity of only 19%.

Similarly, Zhang et al. (38) used pre- and post-contrast T1-weighted MR images, T2 and FLAIR from 87 patients to calculate 285 radiomics features. Interestingly, imaging data from two time points were available so that the authors also investigated feature reproducibility to identify a feature subset with reproducible values. Changes in radiomics features (so-called “delta radiomics”) from one follow-up time point to the other were evaluated and used for differentiation of radiation necrosis and tumor progression. The final model generated by an ensemble classifier had an overall predictive accuracy of 73% and an AUC of 0.73 after cross-validation. Again, no separate dataset for testing was available.

Besides MRI, also amino acid PET images have been used to evaluate radiomics for the differentiation of treatment-related changes from brain metastases recurrence. It has been demonstrated that the evaluation of the time-activity-curves (TAC) that represent the tracer uptake over time is helpful for differentiation of treatment-related changes from brain metastases recurrence (50). However, this requires a time-consuming dynamic FET PET scan of at least 40 min acquisition time or more. Therefore, Lohmann et al. (39) calculated 62 textural parameters on static FET PET scans from 47 patients with MRI findings suspicious for tumor recurrence after radiosurgery. The goal of the study was to investigate whether FET PET radiomics in combination with conventional FET PET parameters could contribute to an improved diagnosis of recurrent tumor. Parameter combinations were investigated using ROC analysis without prior feature selection. The diagnostic accuracy of conventional FET PET parameters was in the range of 81–83% and could be slightly increased to 85% when combined with textural features. Such, FET PET radiomics in combination with conventional PET parameters may have the potential to increase the diagnostic accuracy without the need for a more time-consuming, dynamic FET PET scan. However, no dataset for validation or testing was available.

In a subsequent study, Lohmann et al. (40) investigated the value of combining FET PET and MRI radiomics for the differentiation of treatment-related changes from brain metastases recurrence. Fifty-two patients with newly or progressively contrast-enhancing lesions on MRI after radiotherapy were additionally investigated using FET PET. Prior to feature extraction, images were filtered using three-dimensional wavelet transformation and the LoG filter to enhance edges. Forty-two features were extracted from filtered and unfiltered MR images as well as from summed FET PET images (20–40 min post injection). After feature selection, logistic regression models limited to a maximum of five parameters to avoid over-fitting were generated for the combined PET/MRI features and for each modality separately and validated using cross-validation; no test dataset was available. The highest diagnostic accuracy of 89% (specificity, 96%; sensitivity, 85%) was achieved by the combination of MRI and FET PET features, suggesting that the combined FET PET/MRI radiomics analysis encoded more diagnostic information than either modality alone.

Hotta et al. (41) developed a random forest classifier to differentiate recurrent brain tumor from radiation necrosis based on MET PET in a mixed cohort of 41 patients with brain metastasis (*n* = 21) or glioma (*n* = 20). All patients had been treated with radiotherapy and presented one or more tumor-like lesions on MRI. Forty-two features including conventional and textural features were calculated on summed MET PET images (20–30 min post injection). Afterwards, a random forest classifier was trained to separate radiation necrosis from recurrent brain tumor. The results from the optimized classifier were evaluated using 10-fold cross-validation; no test dataset was available. The most relevant features for classification were identified by using the Gini index (51). The highest diagnostic accuracy with an AUC of 0.98 (specificity, 94%; sensitivity, 90%) was achieved by the radiomics model and outperformed the conventional MET PET parameter evaluation (AUC, 0.73; specificity, 73%; sensitivity, 61%). However, the mixed cohort of gliomas and brain metastases complicates the interpretation of the results.

### Prediction of Brain Metastases Origin

In ~10% of cases, patients are diagnosed with brain metastases without knowing the site of the underlying primary tumor. Conventional MRI usually does not aid the identification of the primary cancer.

The usefulness of radiomics for the prediction of brain metastases origin was investigated by Ortiz-Ramon and colleagues (42). Based on conventional contrast-enhanced T1-weighted MR images of 30 patients with 50 lesions with known primary cancer (27 lung cancer; 23 melanoma), a total of 43 features (3 histogram-based and 40 textural features) were extracted in 2D and 3D, and five predictive models were evaluated using a nested cross-validation scheme. Due to the relatively small number of datasets, no independent test set was available. The highest diagnostic accuracy with an AUC of 0.95 for the differentiation of brain metastases from lung cancer and melanoma was achieved using a model generated by the probabilistic naive Bayes classifier.

In another study of the same group (43), the same question was addressed with a higher number of patients. Contrast-enhanced MRI scans from 38 patients with 67 brain metastases with known primary cancer (27 lung cancer; 23 melanoma; 17 breast cancer) were analyzed. Again, 43 features (3 histogram-based and 40 textural features) were extracted in 2D and 3D. A *z*-score normalization was performed prior to feature selection and a random forest classification within a nested cross-validation structure was applied. The diagnostic accuracy for differentiation of the three primary cancer types had an AUC of 0.87 using 3D texture features. Higher accuracies could be achieved for a one-by-one classification: AUC, 0.96 (lung cancer vs. breast cancer); AUC, 0.96 (lung cancer vs. melanoma). Interestingly, the classification of breast cancer and melanoma brain metastases was unsatisfactory with an AUC of only 0.61. The authors concluded that the volumetric (3D) evaluation of textural features encodes more information and is of higher value for the identification of the primary cancer than 2D features. However, no further model validation was performed.

Kniep et al. (44) also addressed the question of predicting the tumor type in patients with unknown primary lesion at the time of brain metastases diagnosis using MRI radiomics. In that study, 658 brain metastases from 189 patients with known primary cancer were included (151 small cell lung cancer; 225 non-small cell lung cancer; 50 gastrointestinal cancer; 89 melanoma; 143 breast cancer). Imaging data comprised contrast-enhanced and native T1-weighted MRI as well as FLAIR images. Of note, the MR images had been acquired at different MR scanners, thus, the cohort contained heterogenous imaging data. Basic clinical data were combined with 1,423 quantitative image features and evaluated using random forest classification. The final model was validated with model-external cross-validation using an independent training and validation dataset. Furthermore, the results from the classifier were compared with predictions based on conventional image reading by two radiologists. The final model accuracy for classification of all five primary cancer types ranged between an AUC of 0.64 for non-small cell lung cancer brain metastases and an AUC of 0.82 for melanoma brain metastases. The prediction performance was superior to the classification made by two radiologists.

### Differentiation of Brain Metastases From Glioblastoma

Brain metastases and glioblastomas are the two most common malignant brain tumors in adults (52, 53). Importantly, glioblastomas and brain metastases often present similar clinical and imaging characteristics on conventional MRI, resulting in difficult differential diagnosis based on the clinical presentation on standard MRI alone.

Qian et al. (45) addressed this important question using MRI radiomics. A large group of patients (*n* = 412) with untreated brain metastases (*n* = 170) and treatment naive, newly diagnosed glioblastomas (*n* = 242) was divided into a training (*n* = 227) and a test cohort (*n* = 180). Tumors were segmented manually and 1,303 radiomic features were calculated on contrast-enhanced MR images prior to feature selection and model generation. The best classifier that showed a high predictive performance in the test cohort (AUC, 0.90) was a support vector machine algorithm that used least absolute shrinkage and selection operator (LASSO) for feature selection. Also, the classifier showed a better performance than experienced neuroradiologists.

Artzi et al. (46) extracted 760 radiomics features from contrast-enhanced MR images of 439 patients with brain metastases (*n* = 227) or glioblastoma (*n* = 212). After image preprocessing and semi-automatic tumor segmentation using a region-growing algorithm, feature selection, and model generation were performed. Prior to model generation, the datasets were divided into a training and a test cohort in a ratio of 80/20. Interestingly, the authors identified the same support vector machine algorithm as the study by Qian et al. described above, to have the highest predictive performance in the test cohort (AUC, 0.96) for the differentiation of brain metastases from glioblastoma.

Although these studies demonstrated the model performance in an independent test cohort, further external validation is required. However, these two studies nicely demonstrate that radiomics analyses on routinely acquired imaging data already allow the differentiation between brain metastases and glioblastoma with a higher accuracy than experienced neuroradiologists.

### Prediction of Treatment Response

Stereotactic radiosurgery is increasingly used in patients with a limited number and size of brain metastases. However, the treatment response may depend not only on the size but also on the structure of the metastasis which may contain tumor cells and tissue compartments of differing radiosensitivity even within the same histologic type.

Cha et al. (35) tried to predict the response to stereotactic radiosurgery in 89 patients with 110 brain metastases using a deep learning-based radiomics approach by utilizing a convolutional neural networks ensemble radiomics model based on planning CT images. Prior to model generation and evaluation, datasets were randomly assigned to a training, validation, and test cohort. The convolutional neural network learned the classification using training images and labels. The final model was able to predict treatment outcome in the independent test dataset with a high accuracy (AUC, 0.86). The study demonstrates the feasibility of CT-based convolutional neural network radiomics models for the prediction of response to stereotactic radiosurgery also for smaller patient cohorts.

Della Seta et al. (47) demonstrated that sometimes complex radiomics models can be outperformed by a single, conventional imaging feature. Pretreatment contrast-enhanced MR images of 48 patients with singular brain metastases treated with stereotactic radiosurgery were investigated. The subgroup of patients with non-small cell lung cancer brain metastases (*n* = 27) was used to find the ideal cut-off to predict treatment response and the subgroup of patients with melanoma brain metastases were used as validation cohort (*n* = 21). After three-dimensional segmentation of the lesions, tumor volumes and enhancing tumor volumes were determined and the percentage of enhancing tumor volume was calculated. Patients with an enhancing tumor volume of more than 68.6% survived significantly longer (4.9 vs. 10.2 months; *p* = 0.005) and showed significantly longer progression-free survival rates compared to patients with a lower proportion of contrast enhancement. Therefore, the percentage of enhancing tumor volume may be a prognostic imaging marker in patients with singular brain metastases.

Besides stereotactic radiosurgery, immunotherapy has become a valuable treatment option in patients with brain metastases. For example, the advent of immune checkpoint inhibition by antibodies against the programmed cell death protein 1 (PD1; pembrolizumab and nivolumab) or the cytotoxic T lymphocyte antigen 4 (CTLA-4; ipilimumab) resulted in an outcome improvement of patients with melanoma brain metastases. However, there is a subset of patients that do not respond to the immune checkpoint inhibitors and have a poor prognosis. To provide additional diagnostic information over and above what can be derived from anatomical MRI, further imaging biomarkers for the early stratification of patients with melanoma brain metastases according to therapy response are needed.

Bhatia et al. (48) hypothesized that the radiomics analysis of MR images could identify imaging features associated with survival in patients with melanoma brain metastases treated with immune checkpoint inhibitors. Twenty-one radiomics features were extracted from contrast-enhanced MRI scans of 88 patients with 196 melanoma brain metastases. Following manual segmentation, univariate Cox regression was performed for each radiomic feature followed by LASSO regression for dimensionality reduction and multivariate analysis. Several features were found to be associated with an increased overall survival and the mean LoG edge feature best explained the variation in outcome (hazard ratio, 0.68; *p* = 0.001). Unfortunately, no further details about overall survival times were provided. However, no radiomics feature remained statistically significant in the multivariate analysis. Surprisingly, the mean LoG edge feature was confirmed to be a significant predictor of an improved survival in an independent test dataset.

## Conclusions

Taken together, it has to be emphasized that radiomics should be considered as an additional tool to complement established imaging analysis methods and other clinical measures that can be jointly used to make a treatment decision or a final diagnosis with maximum confidence. However, although promising results using radiomics analysis in the field of brain metastases have already been achieved, most studies lack a further validation of the initial results. External validation of the generated models is of high importance and great value to translate radiomics analyses into clinical routine. Another important aspect is the need of standardization of radiomics analysis. In particular, currently self-developed radiomics analysis tools or highly specialized algorithms are predominantly used that may prevent other investigators to reproduce the findings and furthermore limits comparability of the results. In addition, the influence of different scanners and imaging protocols as well as the different preprocessing parameters on the radiomics signatures and the computed models is still not well-understood and needs more attention in future research in order to further promote the translation of radiomics analysis into the clinical workflow.

Notwithstanding, radiomics has a great potential to add valuable additional diagnostic information to many clinical important questions in the field of brain cancer. To overcome the above-mentioned obstacles, respective efforts are currently ongoing.

## Author Contributions

All authors listed have made a substantial, direct and intellectual contribution to the work, and approved it for publication.

### Conflict of Interest

The authors declare that the research was conducted in the absence of any commercial or financial relationships that could be construed as a potential conflict of interest.
